# Impaired growth in rural Gambian infants exposed to aflatoxin: a prospective cohort study

**DOI:** 10.1186/s12889-018-6164-4

**Published:** 2018-11-09

**Authors:** Sinead Watson, Sophie E. Moore, Momodou K. Darboe, Gaoyun Chen, Yu-Kang Tu, Yi-Ting Huang, Kamilla G. Eriksen, Robin M. Bernstein, Andrew M. Prentice, Christopher P. Wild, Ya Xu, Michael N. Routledge, Yun Yun Gong

**Affiliations:** 1 Institute for Global Food Security, School of Biological Sciences, Queen’s University Belfast, Belfast, UK; 2 Division of Women’s Health, King’s College London, London, UK; 3 MRC Unit The Gambia, Serekunda, Gambia; 4 Institute of Epidemiology & Preventive Medicine, College of Public Health, National Taiwan University, Taipei, Taiwan; 5 MRC Elsie Widdowson Laboratory, Cambridge, UK; 6 University of Copenhagen, Copenhagen, Denmark; 7 Department of Anthropology, University of Colorado, Boulder, USA; 8 London School of Hygiene and Tropical Medicine, London, UK; 9 International Agency for Research on Cancer, Lyon, France; 10 School of Medicine, University of Leeds, Leeds, LS2 9JT UK; 11 School of Food Science and Nutrition, University of Leeds, Leeds, UK

**Keywords:** Aflatoxin, Biomarker, Child growth, Insulin-like growth factor, The Gambia

## Abstract

**Background:**

Exposure to aflatoxin, a mycotoxin produced by fungi that commonly contaminates cereal crops across sub-Saharan Africa, has been associated with impaired child growth. We investigated the impact of aflatoxin exposure on the growth of Gambian infants from birth to two years of age, and the impact on insulin-like growth factor (IGF)-axis proteins.

**Methods:**

A subsample (*N* = 374) of infants from the Early Nutrition and Immune Development (ENID) trial (ISRCTN49285450) were included in this study. Aflatoxin-albumin adducts (AF-alb) were measured in blood collected from infants at 6, 12 and 18 months of age. IGF-1 and IGFBP-3 were measured in blood collected at 12 and 18 months. Anthropometric measurements taken at 6, 12, 18 and 24 months of age were converted to z-scores against the WHO reference. The relationship between aflatoxin exposure and growth was analysed using multi-level modelling.

**Results:**

Inverse relationships were observed between lnAF-alb and length-for-age (LAZ), weight-for-age (WAZ), and weight-for-length (WLZ) z-scores from 6 to 18 months of age (β = − 0·04, *P* = 0·015; β = − 0·05, *P* = 0.003; β = − 0·06, *P* = 0·007; respectively). There was an inverse relationship between lnAF-alb at 6 months and change in WLZ between 6 and 12 months (β = − 0·01; *P* = 0·013). LnAF-alb at 12 months was associated with changes in LAZ and infant length between 12 and 18 months of age (β = − 0·01, *P* = 0·003; β = − 0·003, *P* = 0·02; respectively). LnAF-alb at 6 months was associated with IGFBP-3 at 12 months (*r* = − 0·12; *P* = 0·043).

**Conclusions:**

This study found a small but significant effect of aflatoxin exposure on the growth of Gambian infants. This relationship is not apparently explained by aflatoxin induced changes in the IGF-axis.

## Background

Undernutrition and its consequences, including faltered growth, is a major contributor to high mortality rates in children under the age of five years [1]. It can also lead to impaired cognitive ability and reduced school performance, leading to reduced productivity in adult life and economic losses for the country [2]. Although reasonable progress has been made to reduce the global burden of undernutrition in children under five years of age (from 39·6% in 1990 to 23·8% in 2014) the rate of reduction in Africa has progressed more slowly than other regions [3]. Undernutrition is a multifactorial condition; hence, in order to facilitate effective prevention in Africa all underlying risk factors should be identified and targeted.

Exposure to aflatoxin, a mycotoxin produced by fungi that contaminate major cereal crops worldwide, with highest occurrence in hot and humid climates, is a major public health concern due to its carcinogenic [4], immunosuppressive [5] and growth suppressing effects [6]. Populations at highest risk of exposure are those from rural subsistence farming-communities in developing regions, such as in sub-Saharan Africa, where maize and groundnuts are dietary staples and diet variety is poor. In such settings, this high risk of exposure is further compounded by difficulties in the avoidance of contaminated food supplies, limited enforcement of regulatory food standards, and inadequate food storage conditions.

Aflatoxin exposure during foetal development, infancy and early childhood, particularly during the weaning stage when children are gradually introduced to family food, has been associated with low birth weight [7, 8], micronutrient deficiencies [9], growth faltering [10–12], liver damage [13], and immunosuppression [14]. The evidence supporting a causal association between aflatoxin exposure and impaired growth however, is limited. Only a small number of longitudinal studies covering the first 24 months following birth, a critical time period for linear growth, have been conducted in settings where both undernutrition and aflatoxin exposure are prevalent, and the findings among these studies are inconsistent [7, 12, 15].

A number of possible mechanisms by which aflatoxin exposure may cause stunted growth have been proposed, including reduced intestinal absorption of nutrients, and reduced levels of insulin-like growth factor-1 (IGF-1), a peptide hormone that stimulates growth. Aflatoxin causes liver toxicity, which may result in reduced levels of IGF-1 for which the liver is the main site of production [16].

This study aimed to examine the relationship between aflatoxin exposure and growth in Gambian infants from birth to two years of age, and to test the hypothesis that reductions in IGF-axis proteins could be a mechanism for growth impairment. The ENID trial [17] is a randomised trial of nutritional supplementation during pregnancy and infancy on infant immune development. As part of the trial protocol, detailed data on infant growth, feeding practices and morbidity were collected, providing an opportunity to explore how aflatoxin exposure, nutrition and infection interact to reduce growth in children living in an area with high aflatoxin exposure [7, 14, 18].

## Methods

The results from this study embedded within the ENID Trial are reported in accordance with STROBE guidelines.

### Study population

The ENID trial (ISRCTN49285450) primarily examined whether early immune development can be improved through pre-natal and infant nutritional repletion. The trial followed pregnant women and their infants up to one year of age. The ENID-Growth study was an extension of the ENID trial that continued to follow the infants to two years of age. The ENID trial protocol has been described in detail elsewhere [17]. In brief, pregnant women (< 20 weeks gestation) from rural subsistence-farming villages located within The Gambia were recruited in early 2010, and randomised to one of four supplementation groups until delivery: 1) Iron-folate = standard care, 2) multiple micronutrients (MMN), 3) protein-energy (PE) + iron-folate, or 4) PE + MMN. Their infants were then randomised from 6 to 18 months of age to one of two supplementation groups: 1) lipid-based nutritional supplementation (LNS) + MMN, or 2) LNS only.

**Fig. 1:**
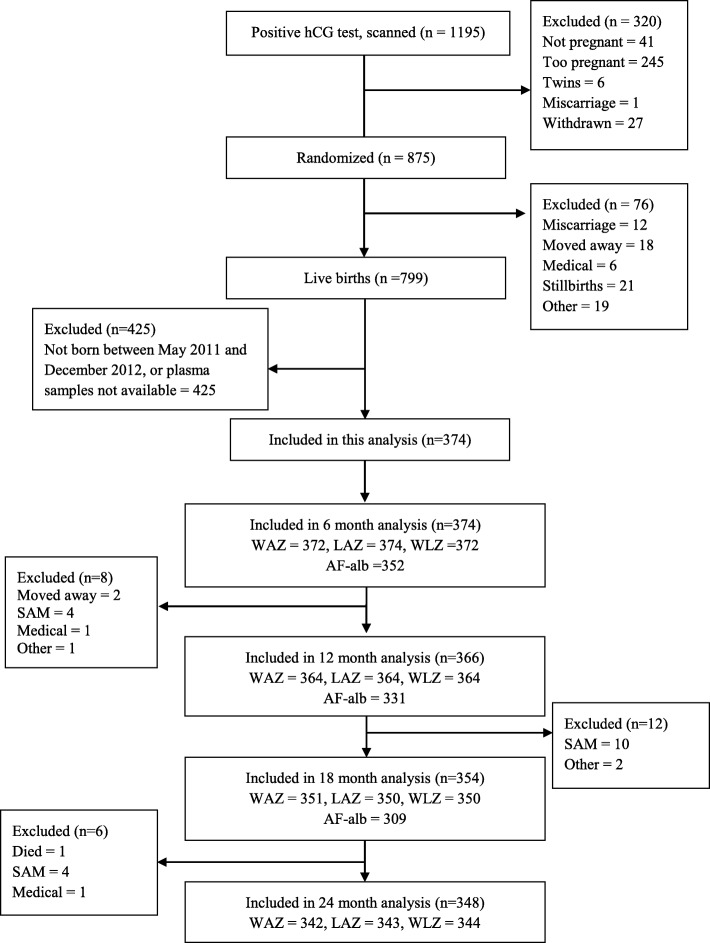
Flow diagram of infants included and excluded in ENID and in this analysis. Abbreviation: SAM, severe acute malnutrition

Infants in the main ENID trial were born between August 2010 and February 2014. For the current sub-study, infants born between May 2011 and December 2012, where plasma samples were available, were included (Fig. 1). All infants received the Expanded Programme on Immunisation as per Gambian government protocol.

### Anthropometric measurements

Anthropometric variables collected at birth (within 72 h of delivery), and at clinic visits when the infants were aged 6, 9, 12, 18, and 24 months were used. Weight was measured to the nearest 0·01 kg using electronic scales and recumbent length was measured to the nearest 0·1 cm using a length board. Growth indicators including length-for-age z-score (LAZ), weight-for-age *z*-score (WAZ), and weight-for-length *z*-score (WLZ) were computed using WHO Anthro software (http://www.who.int/childgrowth/software/en/). Infants were characterised as stunted, wasted, or underweight if they had LAZ, WLZ, and WAZ scores, respectively, below − 2 SD from the median of the WHO reference population.

### Aflatoxin exposure

Blood samples collected at infant ages 6, 12, and 18 months were used to measure aflatoxin-albumin adduct (AF-alb) concentrations at the University of Leeds. AF-alb concentrations in 250 μl plasma samples were measured using a competitive ELISA method [19]. The CV% had to be less than 25% between repeats. The assay’s limit of detection (LOD) was 3 pg/mg albumin. A value of 1·5 pg/mg albumin was assigned to samples with AF-alb concentrations below this limit.

### IGF-axis proteins

Blood samples collected at 12 and 18 months were analysed for IGF-1 and IGF Binding Protein-3 (IGFBP-3) concentrations using IDS-iSYS IGF-1 and IGFBP-3 assays, with the IDS-iSYS Multi-Discipline Automated System (Immunodiagnostic Systems Holdings PLC, UK). The LOD levels for IGF-1and IGFBP-3 assays were 10 ng/mL and 80 ng/mL, respectively. The intra and inter assay CV% for the IGF-1 assay were 3·4 and 6%, and for the IGFBP-3 assay were 2·5% and 5·4%, respectively.

### Infant feeding practice and morbidity

Field assistants visited the infants at home weekly, and administered a morbidity and feeding questionnaire to the primary caregiver (typically the mother). At this visit, the primary caregiver was asked if the infant had experienced any vomiting, diarrhoea, rapid breathing, fever, or cough in the past seven days. At these weekly visits, they were also asked to provide information on breastfeeding practices and the type and frequency of weaning foods the infant consumed.

### Covariates

Household quality, used as an indicator of socio-economic status (SES), was assessed by a questionnaire that collected information on the material of the main structural components (floor, roof and walls) of the house of the mother. The questionnaire was completed in the participants’ homes and were conducted by trained field assistants. For each of the household structural components a list of materials was provided with a scoring guide 1 to 5, with 1 being the lowest score and 5 the highest. For example, for the floors of the house five different types of materials were listed, if earth/sand/mud was used for the floor of the house the lowest score of 1 was entered, if carpet was used the highest score of 5 was entered. A weighted score based on the household materials (multiplied by 0.2 for floor, 0.3 for roof and 0.5 for wall) was then computed for each infant. For analyses infants were then divided into tertiles and classified as belonging to a low, middle or high SES household (cut off at < 2.6 for low; 2.61–3.2 for medium; > 3.21for high).

Other potential confounders included: season of sampling (wet season, June–October, or dry season, November–May), age (months) when non-breast milk foods were introduced (i.e. cessation of exclusive breastfeeding), supplementation group infants were assigned to (LNS + MMN, or LNS only), and incidence of infant diarrhoea and infant morbidity (combined episodes of diarrhoea, vomiting, cough, rapid breathing and fever) in the first two years of life.

### Statistical analysis

Statistical analyses were performed using SPSS version 22·0 (SPSS Inc., Chicago, IL) and STATA version 14 (StataCorp LP).

AF-alb was log transformed (lnAF-alb) and presented as geometric mean, GM (95% CI).

### Relationship between aflatoxin exposure and infant growth

Three separate multilevel linear models (MLM) with maximum likelihood estimation were used to examine the relationship between the repeated measures (at 6, 12, and 18 months of age) of the three infant growth outcomes (LAZ, WLZ and WAZ) and lnAF-alb levels (time-varying covariate also measured at 6, 12, and 18 months). In each model lnAF-alb was modelled as a continuous variable. Measurement occasion was at level one, and individuals at level two. Random effects of the intercept and slopes were allowed, and an unstructured covariance matrix of the random effects was used. All adjusted models included the following covariates: season of sampling (measured at 6, 12 and 18 months), mother’s household quality, supplementation group, and age (months) of introduction of non-breast milk foods. With the assumption that AF-alb value at a given time point represents the average exposure in the previous 6 months (for example AF-alb value at month 12 represents the average exposure between month 6 and month 12) the above MLM models assesses the temporal relationship between infant growth and aflatoxin exposure. Mother’s education was not included as it was not very discriminatory.

Additionally, four separate multilevel linear spline models (MLSM) were used to examine the relationship between lnAF-alb and change in infant growth (WAZ, LAZ, WLZ, and height) at three time intervals (6 to 12 months, 12 to 18 months, and 18 to 24 months). These were added as spline models to allow the slopes to be estimated separately for different observation periods (as the infant growth was not linear during the observation periods). Spline models can also estimate the effect of aflatoxin on different age periods of infant growth. These models use lnAF-alb value at 6, 12 or 18 months as the baseline exposure level of the next 6 months to evaluate its effect on infant growth in the next 6 months. To increase the flexibility in modelling infant growth, a series of linear splines with knots were used to model change in infant growth at the different time intervals. In each model knot points were set at 12 and 18 months, which allowed different linear slopes from 6 to 12 months, 12 to 18 months, and 18 to 24 months, with these slopes varying between individuals. To determine the effect of aflatoxin exposure on change in infant growth for the three time periods, lnAF-alb (modelled as a continuous variable) at 6, 12, and 18 months and their interactions with the linear splines for the three time intervals were then included in the model. If lnAF-alb were related to change in infant growth within a period of time, the interaction would be significantly different from zero.

In the MLSMs, adjustments were made for potential confounders identified from previous studies, these included season of sampling (measured at 6, 12, 18, and 24 months), mother’s household quality, supplementation group, infant morbidity, and age (months) of introduction of non-breast milk foods. Random effects for the baseline body size and the change in infant growth between 6 and 12 months, as indicators of growth scores, measured at three time points (6, 9, and 12 months) within this interval, were included.

### Relationship between aflatoxin exposure and IGF-axis proteins

The associations between lnAF-alb measured at 6, 12, and 18 months of age, and IGF-1 and IGFBP-3 measured at 12 and 18 months of age were examined using Pearson Correlation. Mixed ANOVAs were used to investigate whether change in IGF-1/IGFBP-3 from 12 to 18 months of age was associated with the interaction between the level of AF-alb measured at 12 months of age and time. AF-alb measured at 12 months of age was divided into ‘high exposure’ and ‘low exposure’ by means of a median split, and was the between-subject factor in each model. Time was the within-subject factor in each model, and represented IGF-1/IGFBP-3 measured at 12 and 18 months.

## Results

A total of 374 infants were included in the current sub-study. At 12, 18, and 24 months of age, 366 (98%), 354 (95%), and 348 (93%) of the infants were followed up (Fig. 1). In addition to the participants lost to follow up, ethnicity data was missing for five mothers, SES data for seven and education for four. Mean duration of exclusive breastfeeding was 5·2 ± 1·3 months, and ~ 34% of the infants were exclusively breastfed up to 6 months of age (Table 1). Approximately 8% of the sample had a low birth weight measurement (< 2500 g). Most of the infants’ mothers had no formal education.

**Table 1:** ENID subsample characteristics

Variable	*n*	Mean ± SD
Gender, *n (%)*	374	
Male		192 (51·3)
Female		182 (48·7)
Ethnicity, *n (%)*	348	
Fula		30 (8·6)
Jola		11 (3·2)
Mandinka		304 (87·4)
Other		3 (0·9)
Mothers’ education, *n (%)*	352	
< 1 year formal education		235 (66·8)
> 1 year formal education		117 (33.2)
Birth weight (kg)	335	3·04 ± 0·39
Birth weight categories, *n (%)*	335	
Low (< 2.5 kg)		25 (7·5)
Normal (2.5–3.9 kg)		307 (91·6)
High (≥4.0 kg)		3 (0·9)
Birth length (cm)		50·2 ± 5·0
LAZ at birth		−0·53 ± 0·98
WAZ at birth		−0·57 ± 0·83
WLZ at birth		−0·65 ± 1·11
LAZ at 2 y of age	343	-1·31 ± 0·97
WAZ at 2 y of age	344	-1·33 ± 0·91
WLZ at 2 years	342	−0·93 ± 0·91
Stunted growth at 2 y of age, *n (%)*	343	89 (25·9)
Wasting at 2 y of age, *n (%)*	344	44 (12·9)
Underweight at 2 y of age, *n (%)*	342	84 (24·4)
Age of introduction of non-breast milk foods, *n (%)*	374	
0–3 months		62 (16·6)
4–5 months		186 (49·7)
6 months		126 (33·7)
Diarrhoea episodes (first 2 y of life)		4·3 (3·6)
Total morbidity episodes (first 2 y of life)		13·2 (7·1)
Infant supplementation group, *n (%)*	374	
LNS + MMN		192 (51·3)
LNS only		182 (48·7)

Data are mean ± SD or frequency (percentage). Total morbidity is the combined episodes of diarrhoea, vomiting, rapid breathing, cough and fever in the first 2 years of life. *LAZ* length-for-age z score, *WAZ* weight-for-age z score, *WLZ* weight-for-length z score, *MMN* multi micronutrient, *LNS* lipid-based nutritional supplementation

### Infant growth

**Fig. 2:**
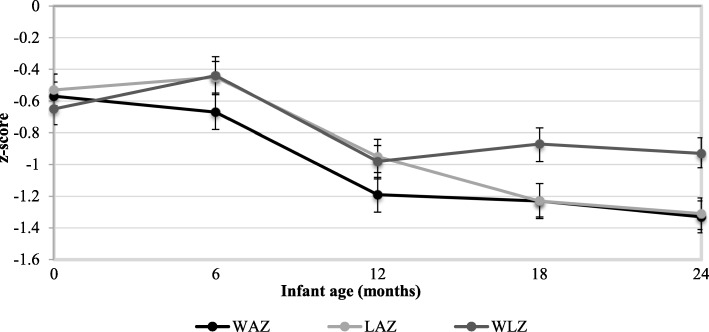
Anthropometric z scores at 0, 6, 12, 18 and 24 months of age. Abbreviations: WAZ, weight for age z-score; LAZ, length for age z-score; and WLZ, weight for length z-score. Values are means ±95% CIs

Mean WAZ, LAZ and WLZ measurements at birth (Fig. 2) were low (− 0·65 ± 1·11, − 0·53 ± 0·98, − 0·57 ± 0·83, respectively). At 2 years of age mean WAZ, LAZ and WLZ scores decreased to − 1·33 ± 0·91, − 1·31 ± 0·97, − 0·93 ± 0·9; respectively (Fig. 2). The proportion of stunting, wasting and underweight increased between six and 24 months of age (5·6% vs. 25·9%, 8·9% vs. 12·9%, and 10·2% vs. 24·4%, respectively).

### Aflatoxin exposure

**Fig. 3:**
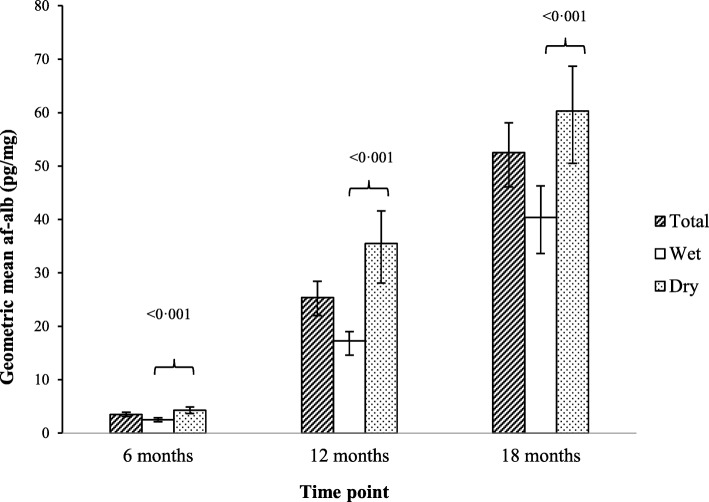
Geometric mean (95% CI) AF-alb concentrations at 6, 12 and 18 months of age, and seasonal differences in AF-alb concentrations. Total = geometric mean AF-alb concentrations; wet = geometric mean AF-alb concentrations measured in blood samples collected during the wet season (June to October); dry = geometric mean AF-alb concentrations measured in blood samples collected during the dry season (November to May). Seasonal differences (wet vs. dry) in lnAF-alb concentrations were analysed using independent samples t-test

At 6, 12 and 18 months of age, approximately 48% (170/352), 98% (325/331), and 99% (307/309) of available plasma samples had detectable AF-alb concentrations (LOD > 3·0 pg/mg), respectively. The higher number of samples below the LOD at 6 months reflects the fact that breast fed infants have lower exposure to aflatoxin, which increases as weaning food is introduced. GM AF-alb concentrations by infant age and season of sampling are presented in Fig. 3. AF-alb concentrations increased as the infants got older (*P* < 0·001), and were higher in samples collected during the dry season than during the wet season.

### Aflatoxin exposure and infant growth

To assess the impact of aflatoxin exposure on infant growth between 6 and 18 months of age we regressed lnAF-alb levels against each z-score at each of the three visits (6, 12, and 18 months) using MLM adjusting for key confounders (Table 2). Inverse relationships were observed over this 12 month period between lnAF-alb and LAZ (β = − 0·04, 95% CI: -0·08, − 0·01, *P* = 0·015) WAZ (β = − 0·05, 95% CI: -0·09, − 0·02, *P* = 0·003) and WLZ (β = − 0·06, 95% CI: -0·10, − 0·02, *P* = 0·007) scores.

**Table 2:** Multilevel linear model of the longitudinal relationship from 6 to 18 months between AF-alb and infant growth z scores

Item	Final model LAZ	Final model WAZ	Final model WLZ
Fixed effects	Coef. (95%CI)	Coef. (95%CI)	Coef. (95%CI)
Intercept	−0·12 (−0·56, 0·32)	−0·83 (−1·28, − 0·39)***	−0·77 (−1·22, − 0·32)***
Time point	−0·33 (− 0·39, − 0·26)***	−0·22 (− 0·28, − 0·16)***	−0·15 (− 0·23, − 0·07)***
lnAF-alb	−0·04 (− 0·08, − 0·01)*	−0·05 (− 0·09, − 0·02)**	−0·06 (− 0·10, − 0·02)**
Season			
Wet	Reference	Reference	Reference
Dry	−0·07 (− 0·14, − 0·00)*	0.16 (0·09, 0·24)***	0·29 (0·20, 0·38)***
Supplementation group			
LNS + MMN	Reference	Reference	Reference
LNS	−0·17 (− 0·37, 0·02)	−0·18 (− 0·38, 0·02)	−0·12 (− 0·32, 0·07)
Breastfeeding			
Age (months) of introduction of non-breast milk foods	−0·01 (− 0·09, 0·07)	0·03 (− 0·05, 0·11)	0·04 (− 0·04, 0·11)
Mother’s household quality			
Low	Reference	Reference	Reference
Med	0·35 (0·13, 0·58)**	0·37 (0·14, 0·60)**	0·27 (0·04, 0·50)*
High	0·23 (−0·03, 0·49)	0·36 (0·10, 0·62)**	0·31 (0·05, 0·57)*
Random effects	Var (95% CI)	Var (95% CI)	Var (95% CI)
Var (Intercept)	0·82 (0·63, 1·06)	0·91 (0·70, 1·18)	1·06 (0·78, 1·44)
Var (Time point)	0·04 (0·02, 0·08)	0·00 (0·00, 0·35)	0·02 (0·00, 0·18)
Cov (Intercept, timepoint)	-0·05 (−0·11, 0·02)	-0·03 (− 0·09, 0·04)	-0·09 (− 0·20, 0·01)
Residual	0·20 (0·17, 0·23)	0·22 (0·18, 0·25)	0·34 (0·29, 0·40)

Analysed using multilevel linear regression modelling with a random intercept and random slope. Final models fitted using maximum likelihood estimation. ***P < 0·001, **P < 0·01, *P < 0·05

As the ENID trial included four maternal supplementation groups as well as the two child supplementation groups we have reanalysed the results to check for any effect of the maternal supplementation groups. No such effect was observed (results not shown).

In separate MLSMs, a significant inverse relationship was observed between lnAF-alb measured at 6 months of age and change in WLZ score between 6 and 12 months of age (β = − 0·01, 95% CI: -0·02, − 0·00; *P* = 0·013). Inverse relationships were found between lnAF-alb measured at 12 months of age and change in LAZ score, and change in length between 12 and 18 months of age (LAZ β = − 0·003, 95% CI: -0·01, − 0·00, *P* = 0·02; length: β = − 0·01, 95% CI: -0·02, 0·00, *P* = 0·003]. No other significant relationships were observed at the other time periods (results not shown).

### Aflatoxin exposure and IGF-axis proteins

IGF-1 and IGFBP-3 concentrations increased significantly between 12 and 18 months of age (Table 3). Both IGF-1 and IGFBP-3 were positively correlated with infant growth measurements at the time point samples were taken (*P* < 0.01).

**Table 3:** Mean IGF-1 and IGFBP-3 concentrations (ng/ml) at age 12 and 18 months

	12 month N	12 month Mean (sd)	18 month N	18 month Mean (sd)	*P* value^a^
IGF1	317	35·7 (14·8)	312	43·2 (17·8)	< 0·001
IGFBP3	292	1670·8 (514·4)	276	1902·6 (619·0)	< 0·001

^a^Mean differences between 12 and 18 month IGF measurements were analysed using paired samples t-test

A negative correlation was observed between lnAF-alb at 6 months and IGFBP-3 at 12 months of age (*r* = − 0·12; *P* = 0·043). No other significant correlations were observed between lnAF-alb levels and IGF-axis proteins. Mixed ANOVA results showed that aflatoxin exposure at 12 months was not associated with change in IGF-1 or IGFBP-3 from 12 to 18 months of age.

## Discussion

The results of this study have confirmed the negative impact of aflatoxin exposure on child growth in children up to 2 years old. We hypothesised based on our previous findings in older children [20] that a reduction in IGF levels could contribute to the mechanism by which this occurs. However, in this population we did not see reduced IGF1 or IGFBP3 associated with aflatoxin exposure.

Growth faltering is common in low and middle income countries [21, 22], and is multifactorial in aetiology. Inadequate dietary intake, infection, early breastfeeding cessation and poverty have all been identified as factors that contribute to faltered growth [23]. The results from this study conducted in The Gambia, covering the period from birth to two years of age, are consistent with the hypothesis that aflatoxin exposure may also be an important factor.

In this population growth faltering and aflatoxin exposure occurred simultaneously. For instance, from 6 to 24 months of age the amount of infants with stunted growth increased fivefold, the amount of wasting doubled and the amount of infants classified as underweight increased threefold. This infant growth pattern is consistently observed within this sub-Sahara African community [24]. Approximately half of the infants’ blood samples had detectable AF-alb concentrations when aged 6 months, and almost all the samples had detectable concentrations when aged 12 and 18 months. These prevalence rates are comparable to those reported in other sub-Sahara African countries, where AF-alb is typically detected in ~ 95% of collected blood samples [25].

Whilst the above evidence shows that aflatoxin exposure and infant growth faltering are both prevalent in this cohort, it does not imply causality; it is possible that both are the consequence of the same circumstances, including poverty and insufficient food intake. Nevertheless, when further analyses were conducted, there was some evidence to suggest that aflatoxin exposure may lie on the causal pathway. For instance, aflatoxin exposure was inversely related to WLZ, WAZ and LAZ scores between 6 and 18 months of age after adjusting for a range of confounders. Furthermore, aflatoxin exposure appeared to temporally proceed impaired infant growth, as infants with higher aflatoxin exposure measured at 6 months of age, demonstrated less gain in WLZ scores between 6 and 12 months, and similarly infants with higher aflatoxin exposure at 12 months of age demonstrated less gain in length and LAZ scores between 12 and 18 months.

Although the size of the effect observed here was not great, these findings contribute to the body of evidence from existing cross-sectional [10, 14, 20, 26–28] and longitudinal studies [7, 12, 29] that have also tested this hypothesis, and have found similar inverse relationships between aflatoxin exposure and LAZ [7, 10, 12, 27–29], WLZ [10, 27, 29] and WAZ [10, 14, 26] scores. There is no doubt that understanding the possible contribution of aflatoxin exposure to child growth impairment is complex, with many other potential contributing factors. In our longitudinal study conducted in Tanzania (infants aged 6–14 months) [15], a trend in lower growth over 12 months in children with higher AF-alb did not reach statistical significance, but we did find a significant inverse association between exposure to another mycotoxin that contaminates maize, fumonisin, and LAZ scores. Mean AF-alb concentrations observed over the three sampling time points in that particular study, however, were lower than those observed in this current study, consistent with higher levels of exposure that have been associated with groundnut intake (more common in Gambia) versus maize intake, elsewhere [30].

Notably, the associations between aflatoxin and growth impairment observed in the current study remained after controlling for a range of important confounders including mother’s household quality, an indicator of SES. Low SES is related to inadequate dietary intake and infectious diseases, which consequently can lead to impaired linear growth during childhood. There is also evidence to suggest its association with higher aflatoxin exposure. For instance, Leroy et al. [31] found an array of socioeconomic determinants associated with lower aflatoxin exposure levels in rural Kenyan women, including higher levels of education, land ownership, food security, higher household expenditure and use of fertiliser. While the current study did not find a significant association between mother’s household quality and aflatoxin exposure, we have not tested how well these measurements of household quality are associated with wealth.

To further elucidate the relationship between aflatoxin exposure and child growth impairment it is essential that the molecular and biological mechanisms by which aflatoxin exposure causes impaired growth be identified. It has been proposed that alteration of the growth hormone-IGF system by aflatoxin exposure, possibly due to protein synthesis, liver toxicity or DNA methylation, could be a potential pathway. In an earlier study of older Kenyan children [20], it was estimated that 16% of the effect of aflatoxin being associated with reduced child height could be explained by reduced IGF1/IGFBP-3 levels. In this current study there were no significant associations between IGF1 and AF-alb concentrations, but an inverse relationship was observed between AF-alb levels at 6 months of age and IGFBP-3 concentrations at age 12 months. There are a number of differences between the two studies, most notably that the prior study was cross-sectional, the population were adolescents and that a different method was used to quantify the IGF-axis proteins. Further research is, therefore, warranted to determine if alteration in IGF-axis by aflatoxin exposure is a mechanistic pathway for growth stunting, particularly during the critical 24 months after birth.

The strengths of this study include the large representative sample that is well characterised owing to the data collected longitudinally on morbidity, feeding practices and infant growth. Also objective measurements of aflatoxin exposure and growth were used which enhances the validity of the results. The main limitation of this research is that an observational study design was used, which is susceptible to potential bias, including response bias (specifically social desirability bias) for the household quality questionnaire and recall bias for the morbidity and feeding questionnaires. Since outcomes are measured as sectional events and initiation of exposure before outcome onset cannot be clearly established there is also potential temporality bias. Although this sub-study was from within a nutrition intervention trial, the population sampled was representative of the local population as all women within the area who became pregnant during the trial period were available for recruitment (with specific eligibility exceptions [17]), and were randomised to the intervention on recruitment. Furthermore, the WAZ, LAZ and WLZ scores (6, 12, 18 and 24 months) did not significantly differ according to infant supplementation group, so we are confident that the nutrition intervention did not influence our findings.

## Conclusions

This study of Gambian infants, covering the first two years following birth, found that aflatoxin exposure was associated with impaired growth. The findings contribute to a body of evidence that suggests aflatoxin may be an underlying determinant of impaired child growth. Further research, however, is required to identify the biological mechanistic pathways, and to design and implement intervention studies that target aflatoxin exposure alongside child undernutrition.

## Abbreviations

AF-Alb: Aflatoxin albumin adduct
GM: Geometric mean
IGF-1: Insulin-like growth factor 1
IGF-BP3: Insulin-like growth factor binding protein 3
LAZ: Length for age Z score
LNS: Lipid-based nutritional supplementation
LOD: Limit of detection
MLM: Multilevel modelling
MLSM: Multilevel linear spline models
MMN: Multiple micronutrients
SES: Social economic status
WAZ: Weight for age Z score
WLZ: Weight for length Z score
